# An ordinal severity scale for COVID-19 retrospective studies using Electronic Health Record data

**DOI:** 10.1093/jamiaopen/ooac066

**Published:** 2022-07-09

**Authors:** Maryam Khodaverdi, Bradley S Price, J Zachary Porterfield, H Timothy Bunnell, Michael T Vest, Alfred Jerrod Anzalone, Jeremy Harper, Wes D Kimble, Hamidreza Moradi, Brian Hendricks, Susan L Santangelo, Sally L Hodder, N3C Consortium Collaborators, Christopher G Chute, Melissa A Haendel, Anita Walden

**Affiliations:** West Virginia Clinical and Translational Sciences Institute, Morgantown, West Virginia, USA; West Virginia Clinical and Translational Sciences Institute, Morgantown, West Virginia, USA; Department of Management Information Systems, West Virginia University, Morgantown, West Virginia, USA; Department of Medicine, University of Kentucky, Lexington, Kentucky, USA; Biomedical Research Informatics Center, Nemours Children's Health, Wilmington, Delaware, USA; Section of Pulmonary and Critical Care Medicine, Christiana Care Health System, Newark, Delaware, USA; Department of Medicine, Sidney Kimmel College of Medicine, Philadelphia, Pennsylvania, USA; Department of Neurological Sciences, University of Nebraska Medical Center, Omaha, Nebraska, USA; Owl Health Works LLC, Indianapolis, Indiana, USA; West Virginia Clinical and Translational Sciences Institute, Morgantown, West Virginia, USA; Department of Data Science, University of Mississippi Medical Center, Jackson, Mississippi, USA; Department of Epidemiology, West Virginia University, Morgantown, West Virginia, USA; Center for Psychiatric Research, Maine Medical Center Research Institute, and Maine Medical Center, Portland, Maine, USA; Department of Psychiatry, Tufts University School of Medicine, Boston, Massachusetts, USA; West Virginia Clinical and Translational Sciences Institute, Morgantown, West Virginia, USA

**Keywords:** COVID-19 ordinal scale, Electronic Health Record, National COVID Cohort Collaborative, N3C

## Abstract

**Objectives:**

Although the World Health Organization (WHO) Clinical Progression Scale for COVID-19 is useful in prospective clinical trials, it cannot be effectively used with retrospective Electronic Health Record (EHR) datasets. Modifying the existing WHO Clinical Progression Scale, we developed an ordinal severity scale (OS) and assessed its usefulness in the analyses of COVID-19 patient outcomes using retrospective EHR data.

**Materials and Methods:**

An OS was developed to assign COVID-19 disease severity using the Observational Medical Outcomes Partnership common data model within the National COVID Cohort Collaborative (N3C) data enclave. We then evaluated usefulness of the developed OS using heterogenous EHR data from January 2020 to October 2021 submitted to N3C by 63 healthcare organizations across the United States. Principal component analysis (PCA) was employed to characterize changes in disease severity among patients during the 28-day period following COVID-19 diagnosis.

**Results:**

The data set used in this analysis consists of 2 880 456 patients. PCA of the day-to-day variation in OS levels over the totality of the 28-day period revealed contrasting patterns of variation in disease severity within the first and second 14 days and illustrated the importance of evaluation over the full 28-day period.

**Discussion:**

An OS with well-defined, robust features, based on discrete EHR data elements, is useful for assessments of COVID-19 patient outcomes, providing insights on the progression of COVID-19 disease severity over time.

**Conclusions:**

The OS provides a framework that can facilitate better understanding of the course of acute COVID-19, informing clinical decision-making and resource allocation.

HIGHLIGHTS We developed a generalizable COVID-19 severity scale to facilitate research on COVID-19 patient outcomes using real-world data.

Electronic Health Record data on 2 880 456 SARS-CoV-2-infected patients from 63 health centers across the United States were examined using National COVID Cohort Collaborative. Concept sets were identified and validated using standard medical terminologies necessary to assign a disease severity to each patient.

Patterns of changes in disease severity among patients were characterized during the 28-day period following a SARS-CoV-2 diagnosis.

## INTRODUCTION

Infection with the novel coronavirus (SARS-CoV-2) is associated with a spectrum of symptomatology ranging from no symptoms to severe illness and death. The ability to measure the severity of COVID-19 plays an important role in evaluating therapeutic outcomes. Multiple metrics have been used to measure COVID-19 patient outcomes, including hospital admission, intensive care unit (ICU) admission, length of ICU stay, requirement for mechanical ventilation, and death.

In 2020, the World Health Organization (WHO) convened a special committee to create the WHO clinical progression scale for purposes of measuring COVID-19 severity over time.^1^ This scale has been widely used in COVID-19 prospective clinical trials and cohort studies.^2^^,^^3^ However, the WHO scale is less useful for studies in which only discrete Electronic Health Record (EHR) data are available as some of the features used to assign the WHO scale are not recorded in the EHR, primarily because they are not recorded as discrete data elements, do not adhere to interoperable data standards, or cannot be identified or interpreted with fidelity. Evaluation of large EHR databases, such as the National COVID Cohort Collaborative (N3C), containing data on millions of persons with a diagnosis of SARS-CoV-2 infection, are now available and are potentially an invaluable source for analyses to inform the understanding of COVID-19 patient outcomes among various patient subsets. However, assigning illness severity can be challenging when data are derived entirely from EHRs. A clinical severity scale appropriate for EHR analyses that recapitulates the patterns and clinical relevance of the WHO scale is needed.

Other scales for studying COVID-19 severity in EHR data have been proposed. One such scale from the Consortium for Clinical Characterization of COVID-19 by EHR (4CE) developed a severity phenotype for COVID-19 using EHR data coded on the i2b2 ontology in the Accrual to Clinical Trials network.^4^^,^^5^ The 4CE severity phenotype applies to hospitalized patients only and was developed as a proxy for poor patient outcomes. While this severity scale provides a model for potential use, it has not been used widely outside the i2b2 network. Since i2b2 is not as widely used as other data models, deploying it for use in other research data networks, which rely on different terminology standards, would take significant modification.

A second scale is an N3C-specific assessment tool measuring the most severe clinical outcomes post-SARS-CoV-2 infection.^6^ This pivotal work characterizes the N3C clinical cohort and establishes an outcome-based severity measure. Since this is an outcome scale, it does not allow for the establishment of a baseline severity at admission or at a point in time during a hospital visit.

The objective of this study is to develop a scalable, time-sensitive ordinal scale (OS) to facilitate robust, clinically relevant evaluation of COVID-19 disease progression and patient outcomes evaluation for use in multi-site, real-world studies using EHR data. In this report, we describe the developed OS and assess its usefulness in describing COVID-19 outcomes in the N3C dataset.

## MATERIALS AND METHODS

### Data source

N3C includes data from 63 sites across the United States creating a comprehensive SARS-CoV-2 clinical data repository. N3C combines EHR data contributed by various dissimilar networks, managing, and sharing the harmonized data with the goal of fast-tracking insights to assist in addressing the pandemic.^6–8^ N3C collects longitudinal EHR data on all patients from the 63 submitting institutions with a confirmed COVID-19 diagnostic code (∼22% of all positive patients in N3C) or a confirmed positive SARS-CoV-2 laboratory test (∼78% of all positive patients in N3C) as well as SARS-CoV-2 negative patients after January 1, 2020.^9^ Data available in N3C include demographic, clinical characteristics, diagnostic studies, medication, and laboratory values, including entries from up to 2 years before a SARS-CoV-2 laboratory test or diagnosis. Source data for N3C are harmonized into the Observational Medical Outcomes Partnership (OMOP) Common Data Model (CDM) which was designed by Observational Health Data Sciences and Informatics (OHDSI) specifically for conducting research and analyses, facilitating patient classification in an interoperable fashion from source data that are primarily built to support healthcare operational requirements.^10–12^ OHDSI provides different tools to verify completeness, conformance, and plausibility of data when mapping data to OMOP CDM.^12^^,^^13^ In addition, N3C developed a pipeline for ingesting, harmonizing, and centralizing data using different federated common data models as well as automated and manual data quality testing procedures using several heuristic approaches.^14^ The N3C Data Ingestion and Harmonization process involves substantive unit harmonization and value set mappings that have been previously validated with 12% data loss between data models noted.^15^

This study received Institutional Review Board approval and was reviewed and approved by the N3C Data Access Committee.

### Ordinal severity scale

Within OMOP, any piece of information including clinical events is mapped and linked to the appropriate standard concept. All relevant concepts within N3C were independently reviewed and deemed appropriate for inclusion by 2 physicians and are available on our project GitHub repository.^16^ We investigated a set of concepts that were deemed to be unique and to define patient severity levels, as well as a “best string search” strategy for finding future concepts. A final concept set was created based on the clinical indication and number of times it was included in the database. Supplementary Tables SA1–SA3 present an analysis of the most frequent concepts used in our developed OS by week after SARS-CoV-2 diagnosis.

Based on the concept sets, 6 levels of clinical severity were developed corresponding to odd integers between 1 and 11. It is likely that a patient can be identified with concepts associated with multiple levels of the OS simultaneously in which patient’s OS is assigned as the most severe (highest score) over a given time period of interest, for example, a day for daily OS or a 7-day window for weekly OS. The selected concepts were queried for each patient within the first 28 days of their first COVID-19 diagnosis.

### Data analysis

Multiple analyses were used to test the utility of the OS including a principle component analysis (PCA) that characterizes patterns of variation in OS levels over the course of the 28-day period. PCA extracts the most important information from the data and analyzes the structure of the cases and daily OS of our data by utilizing the spectral decomposition of the data. The results of PCA create new orthogonal components or dimensions that summarize the variability in the data (eg, weighted averages), which are useful as they can be interpreted in the context of the data. The first new variable recommended by PCA, PC1, is the variable that explains the largest proportion of variability in the data set; the second new variable recommended by PCA, PC2, is the variable, independent of PC1, that explains the second most variation in the data.^17^^,^^18^ For comparison purposes, the data were first centered and scaled before implementing PCA. In the context of this analysis, PC1 and PC2 represent summary statistics that indicate daily changes of a patient’s OS levels, indicating major change points in the OS.

Most commonly, PCA is applied to continuous variables and uses the patterns of correlation or covariance among the variables to derive a set of orthogonal dimensions as weighted combinations of all original variables associated with each patient. Although the variables in the current dataset are ordinal rather than continuous, they represent a rank ordering of disease severity. Standard correlation measures using ranks provide a Spearman rather than a Pearson correlation coefficient and consequently represent a valid use of the PCA.

## RESULTS

### OS description

**Figure 1.**
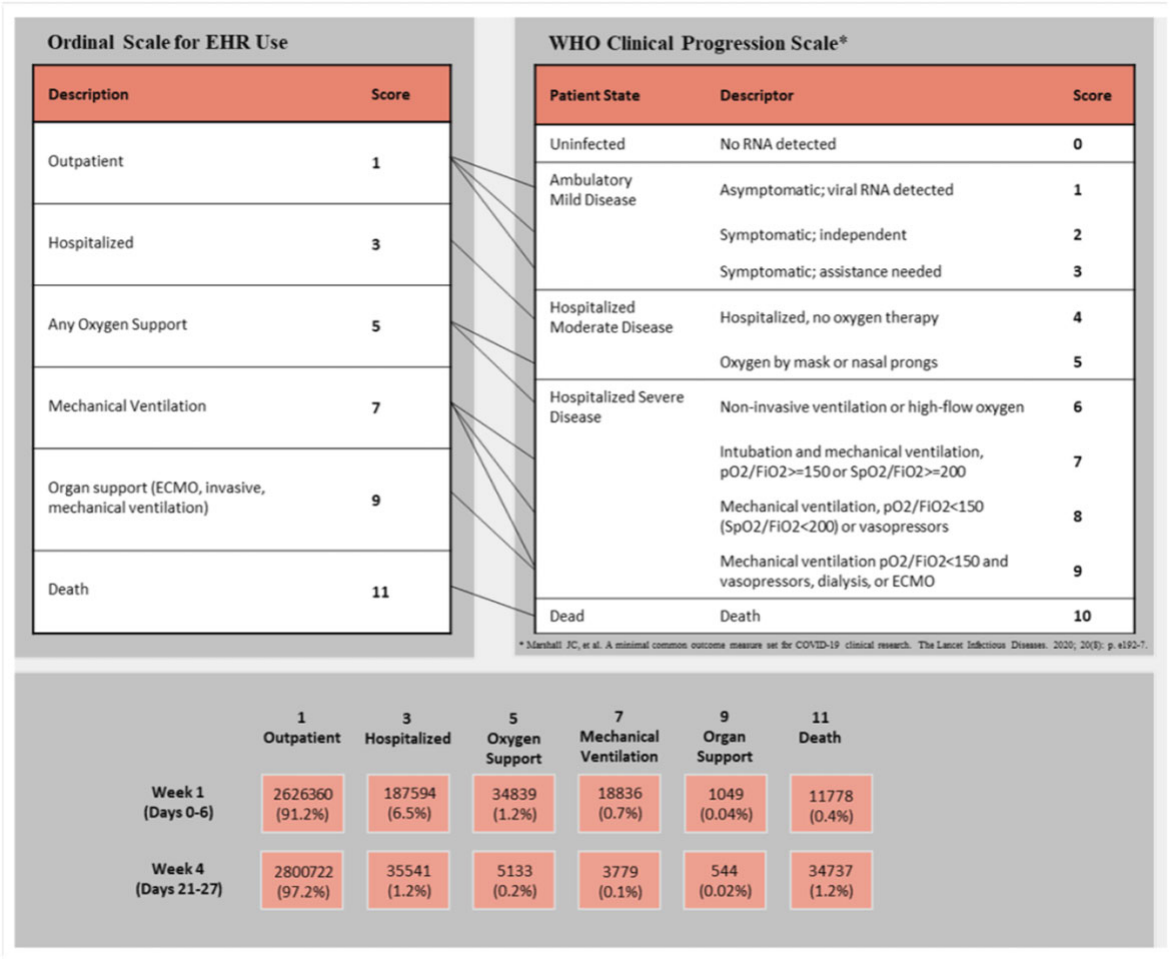
Ordinal scale for EHR use compared to WHO Clinical Progression Scale. HER: Electronic Health Record; WHO: World Health Organization.

The 11-point, 6-level, scale of SARS-CoV-2 clinical severity is shown in Figure 1. An OS score of 1 indicates outpatient status while OS 3 indicates hospital admission. An OS of 5 indicates use of supplemental oxygen, including high-flow oxygen, that may incorporate multiple EHR codes, including nonmechanical respiratory ventilation, assistance with respiratory ventilation, oxygen delivered as a medication, and oxygen administration by nasal cannula (Supplementary Table SA1). OS 7 denotes requirement for mechanical ventilation and incorporates EHR codes including respiratory ventilation, intubation, insertion of endotracheal airway into trachea, insertion of airway into mouth and throat, nasopharynx, or trachea (Supplementary Table SA2). OS 9 indicates requirement for extracorporeal membrane oxygenation (ECMO) (Supplementary Table SA3). OS 11 indicates death.

### Patient sample

Our study includes EHR data on 2 880 456 patients with a diagnosis of SARS-CoV-2 infection submitted from 63 institutions from January 1, 2020, through October 1, 2021. Cohort demographics include the following: 54% female, 13% black or African American, 13% Hispanic or Latino, and 17% >65 years of age (Table 1). On the day of SARS-CoV-2 diagnosis, 93% of the patient sample were outpatients (OS 1) and 6% were admitted to the hospital (OS 3) (Table 1).

**Table 1.** Baseline characteristics of the study population (*n* = 2 880 456)

Characteristic	*N* (%)
Gender	
Female	1 545 048 (54%)
Male	1 289 637 (45%)
Missing/unknown	45 771 (1.6%)
Age	
<18	345 541 (12%)
18-29	548 921 (19%)
30-49	869 560 (30%)
50-64	629 905 (22%)
≥65	484 213 (17%)
Race	
White	1 573 278 (55%)
Black/African American	363 834 (13%)
Asian	63 527 (2.2%)
Hawaiian	6795 (0.2%)
Other/unknown	873 022 (30%)
Ethnicity	
Not Hispanic Latino	1 887 044 (66%)
Hispanic or Latino	378 843 (13%)
Missing/unknown	614 569 (21%)
Day 1 OS	
OS 1	2 683 245 (93%)
OS 3	159 875 (6%)
OS 5	21 797 (0.8%)
OS 7	12 902 (0.4%)
OS 9	643 (0.02%)
OS 11	1994 (0.07%)

OS: Ordinal scale.

**Figure 2.**
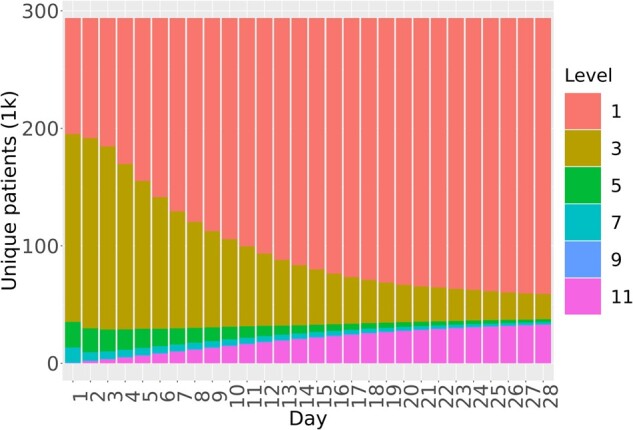
Distribution of OS for hospitalized patients over the 28 days after SARS-CoV-2 diagnosis. OS Level 1: Outpatient, OS Level 3: Hospitalized, OS Level 5: Oxygen support, OS Level 7: Mechanical ventilator, OS Level 9: Organ support, OS Level 11: Death.

Figure 2 shows the distribution of OS level for the study population throughout the 28-day period following SARS-CoV-2 diagnosis, but only for those patients who were hospitalized at least once within the 28 days. As this figure illustrates, proximal to diagnosis, OS level 3 patients predominate, but the number of patients who are not hospitalized increases rapidly during the first 14 days. The number of patients in each of the intermediate OS levels decreases throughout the 28-day interval, while the number of patients at OS level 1 (not hospitalized) increases. The number of patients with OS 11 or death decreases through 28-day interval, but cumulative death, shown in Figure 2, increases.

### OS variation by week

**Figure 3.**
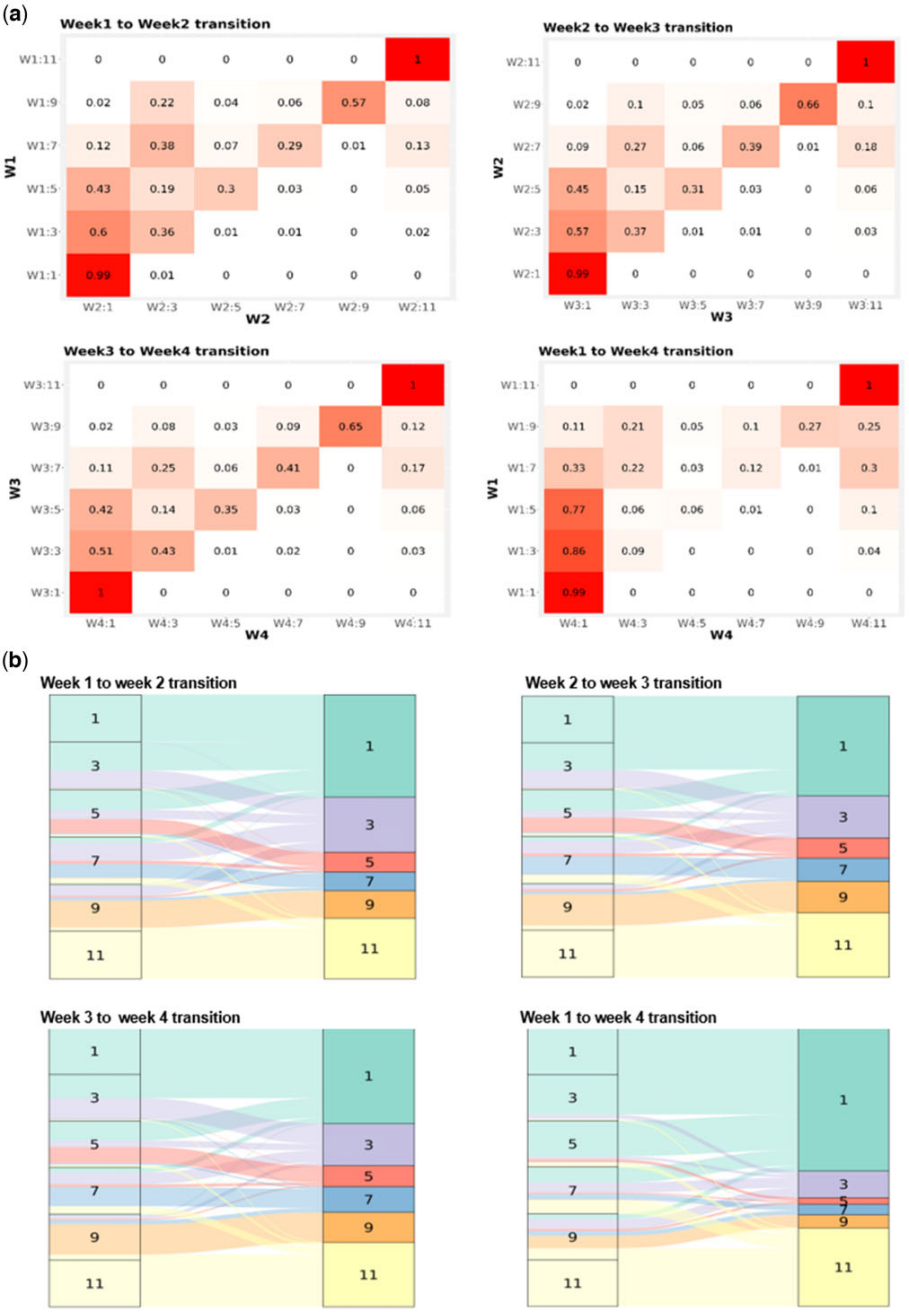
Heatmap (A) and ribbon chart (B) of the proportion of patients that transition OS scores by week. The first number following W (week) represents the week since diagnosis (ie, week 1, 2, etc.); the second number represents the sample at given OS. Each cell in heat map represents probability of a patient starting at OS on vertical axis transitioning to OS level on the horizontal assess. Ribbon charts show transitions right to left, and color coded based on outcome in latest week.

To further characterize transitions from one OS level to another during the 4-week period following SARS-CoV-2 diagnosis, we examined the proportion of individuals who move from one level of OS to another on a week-by-week basis. Figure 3 presents a heat map of the transition matrix for selected week-to-week changes. Each cell in the heat map represents the probability of a patient at the OS of the starting week (vertical axis) transitioning to various levels of OS during the following week (horizontal axis). For example, the probability that patients with OS score 1 in week 1 (W1:1) stay at OS 1 during week 2 (W2:1) is 0.99. The results show that most patients do not need hospitalization and most of those who are hospitalized transition to lower OS scores (ie, lower disease severity) over time. Figure 3 also demonstrates that more severely ill individuals had persistently higher OS scores than less severely ill patients who tended to resolve to a lower OS designation more quickly. Table 2 presents the length of hospital stay and the 28-day mortality rate stratified by the maximal OS level reached in week 1 OS level and day 1 OS level, respectively. The results show that patients with higher OS level had longer hospital stays and higher mortality rates. Supplementary Figure SA1 shows the weekly transition and outcome of patients who were hospitalized during the 4-week period color-coded based on week 1 OS score.

**Table 2.** Twenty-eight-day mortality rate and length of stay by week 1 OS level

	Mortality rate (%)	Length of stay (days)
Week 1 OS		
OS 1	0.2	0.1
OS 3	4.1	7.2
OS 5	10.0	8.8
OS 7	29.6	16.7
OS 9	24.9	22.2
Day 1 OS		
OS 1	0.4	0.2
OS 3	8	8.1
OS 5	14.8	8.8
OS 7	36.2	15
OS 9	32.7	20.3

OS: Ordinal scale.

The weekly transition heat maps (Figure 3) also demonstrate that the transition matrix from week 2 to week 3 is similar to that of week 3 to week 4, but both differ from the transitions found in week 1 to week 2. That is, there are fewer transitions in the OS observed from week 2 to week 3 and from week 3 to week 4 when compared to the number of transitions observed between week 1 and week 2. This observation may indicate that the most impactful period for improving patient outcomes is generally from week 1 to week 2.

### Daily OS over 28 days

**Figure 4.**
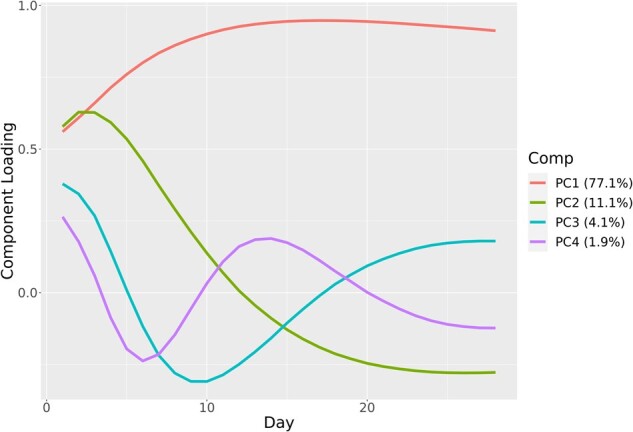
Variation of the first 4 principal components by daily OS. PC1: Overall disease severity over 28 days, PC2: Contrasts days 1–12 compared to days 13–28, PC3: Contrasts week 1 compared to week 2, PC4: Rapid changes within day 1–12, OS: Ordinal scale.

Despite the purely “ordinal” nature of the OS, we sought to characterize the per-patient variation over time to better understand changes in the OS over time, using PCA. As is common in PCA, the data were first scaled. Figure 4 shows the 28-day weight vectors for each of the first 4 principal components (PCs). These 4 components combined to account for 94.2% of the variance in per-patient OS level over the 28-day interval.

The first PC (accounting for 77.1% of the variance) is a weighted average of a patient’s OS during the 28-day period. The weights that are all positive on the daily OS place higher emphasis on the latter 2-week period in the OS, typically when patients do not have many changes in severity. The second PC (accounting for 11.1% of the variance) can be interpreted as contrasting the average OS over days 1–12 with the average OS in days 13–28. Patients with large or more positive values for PC2 will be those who tend to have entered the hospital with higher OS levels in approximately the first 2 weeks and who improved to lower OS levels in the final 2 weeks. Conversely, patients with smaller or negative values will be those with lower OS levels initially and who did not improve or moved to higher OS levels in the second 2-week period. The other 2 PCs, that is, PC3 and PC4, have more complex patterns with respect to OS scores over time, and both captured distinct rapid changes in OS level over the first 2 weeks. It is notable that all components converge toward moderate stable weights by the end of the 28-day interval.

**Figure 5.**
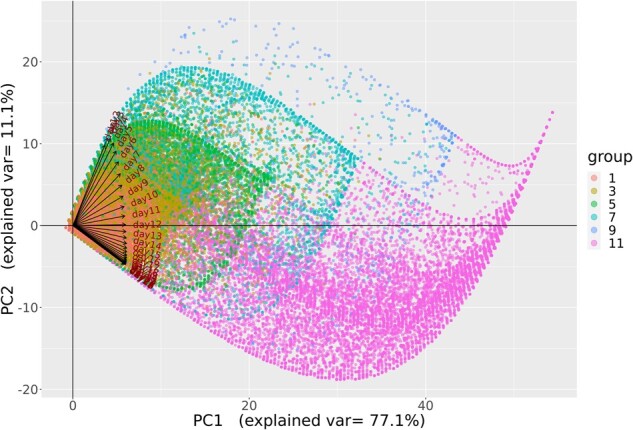
PC1 versus PC2 grouped by maximum OS. OS: Ordinal scale; PC: Principal component.

**Figure 6.**
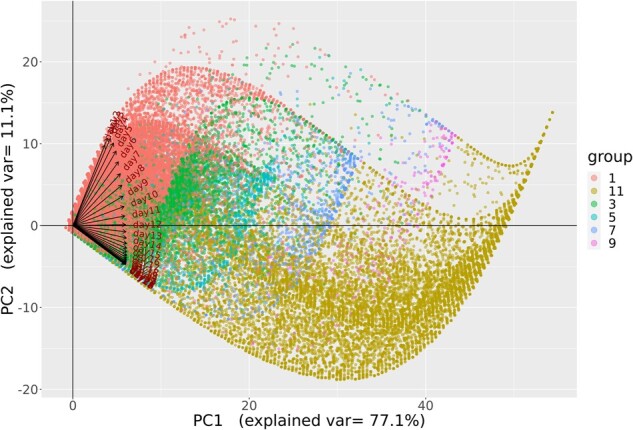
PC1 versus PC2 grouped by final OS after 28-day period. OS: Ordinal scale; PC: Principal component.

Figures 5 and 6 present visual representations of this analysis where the lines show the daily weights for each variable on PC1 and PC2. As demonstrated, the days of week 1 have greater projection on the *y*-axis and smaller on the *x*-axis, meaning that early days have greater contribution to PC2 and smaller to PC1, compared to other days. After week 1, the days have nearly the same value on the *x*-axis, showing that their contribution to PC1 is almost equal, while the days before and after day 12 mirror each other on PC2.

The color coding represents the maximum OS value of the 28-day period and the final value of the 28-day period in Figures 5 and 6, respectively. Patients with OS 1 or OS 3 tend to have lower PC1 compared to patients with OS 5 or 7 in both figures. Similarly, patients with OS 11 tend to have lower PC2 compared to OS 9. These figures provide confirmation of the meaning of each of the variables suggested by PCs, which detail the change in variability over time. Specifically, the results show how the OS can be used to define multiple statistics to summarize the patient's severity during the 28-day period.

The PCA also provides confirmation of the difference in variability over the 28-day period between the first 12 days and the subsequent 16 days when considering the changes in the OS. Finally, PCA provides an example of the flexibility of the OS to be treated as a continuous metric for analysis and provides insights into the changes in disease severity over the 28-day period.

## DISCUSSION

We demonstrated the feasibility and utility of a clinically meaningful OS to measure COVID19 severity that is practical to use with EHR data. Similarities exist between our OS for EHR data analyses and the WHO Clinical Progression Scale, developed for use with prospective studies. The WHO clinical progression scale has 3 levels of clinical severity that are applied to outpatients: (1) asymptomatic, viral RNA detected; (2) symptomatic, independent; and (3) symptomatic, assistance needed.^1^ As EHR data often have a relative paucity of detailed outpatient data, our OS scale collapses all outpatients into 1 category. While this large group of patients undoubtedly has a range of clinical symptoms, EHR data do not reliably distinguish between various SARS-CoV-2 severity states among outpatients.

Similarly, accurately identifying degrees of oxygen supplementation (mask, nasal cannula, noninvasive ventilation, or high-flow oxygen) using EHR data is difficult and not considered to be robust, particularly for harmonized datasets such as those in the N3C, given the variability in institutional coding practices. Oxygen orders are often entered irrespective of need and degree of oxygen supplementation and do not always strongly correlate with actual use. In addition, the concept codes for noninvasive ventilation are widely variable and it is difficult to separate supportive use for oxygenation versus continuation of chronically used therapy for sleep apnea or other purposes. As such we combined WHO score 5 (oxygen by mask or nasal prongs) with WHO OS 6 (noninvasive ventilation or high-flow oxygen) into our OS score 5.

With regard to OS 9, both organ support and renal replacement therapy are represented by a significant diversity of EHR codes; therefore, these were thought to be unreliable for identification in the N3C database. By comparison, ECMO was found to have more straightforward concept codes and thus were selected as the markers for additional organ support distinguishing this group (OS = 9).

A notable difference between our OS and that of the WHO is the inclusion of vasopressors. In the WHO scale, a score of 8 denotes mechanical ventilation OR vasopressors and a score of 9 denotes mechanical ventilation AND vasopressors, dialysis, or ECMO.^1^ Originally, an individual receiving pressors, regardless of the presence or absence of other treatment modalities (eg, oxygen therapy) was classified as OS 9. However, we assessed the frequency of vasopressor use across OS levels, and much to our surprise, vasopressors were used in each OS level from 3 to 9. We found that there were individuals who received pressors but did not receive any oxygen. Not surprisingly, individuals receiving pressors but not ECMO or mechanical ventilation had a lower risk of dying. We, therefore, have excluded vasopressors as a criteria in our OS.

The use of odd numbers in the proposed OS has 2 main justifications. The first is an acknowledgment that the proposed OS combines levels of the original WHO scale in its development. The second justification is that the use of odd numbers in the proposed OS allows researchers to add levels of severity based on the study of interest at intermediate steps without redefining the scale itself. This approach marries the need to categorize patient severity along with the ability to introduce greater granularity. For instance, an OS 4 could be created with all hospitalized patients who were on vasopressors (but not receiving oxygen supplementation). OS 6 could include all patients who were receiving oxygen and vasopressors, and OS 8 and OS 10 follow suit for mechanical ventilation and organ support. Finally, OS 0 could also be created to identify uninfected patients.

Strengths of this approach are that changes in OS are used as a metric for overall worsening or improvement in the severity of disease; Figure 3 shows a higher chance of dying as OS level increase. OS can also be used to track patient cohorts at any level of illness and facilitate assessment of various treatment modalities coded in EHR data. EHR datasets, such as those that are harmonized in N3C, include extremely large numbers of patients, which facilitate the assessment of outcomes in subsets of patients. Many COVID-19 therapeutic trials are underpowered to assess differences in outcomes for specific patient subgroups.^2^^,^^19–21^ However, EHR real-world data may meet this need. It is acknowledged that retrospective, observational data have limitations such as unrealized confounding; however, the very large sample size of the N3C cohort enables discoveries that may not be possible with prospective clinical trials. As such, robust EHR real-world data analyses complement clinical trial data.

Our analyses demonstrate differences between outcomes from the first and second 2-week periods following a SARS-CoV-2 diagnosis. Specifically, our finding that OS level changes occurring during the first 2 weeks after diagnosis are more frequent compared to the third and fourth weeks after diagnosis suggests a time period when therapeutic interventions might be most effective. We also show how the use of the OS as an ordered variable that ranks disease severity can be used to analyze daily variation to better understand the progression of clinical severity among COVID-19-positive patients over time. Furthermore, results of the PCA provide deeper insights and are similar to results of the analysis of transitions using weekly maximum OS. The results of the PCA illustrate the utility of the proposed OS to develop summary statistics of patient severity over a given time period. The consistency of results between the 2 analyses demonstrates the flexibility of the OS in multiple settings. For example, when there are concerns regarding daily variability secondary to data quality issues, one may feel confident in using a weekly analysis.

A benefit of the proposed OS is that it quantifies COVID-19 disease severity at any given time point, while not restricting the set of analytic tools to those only associated with ordinal data. The proposed OS is only considered ordinal at a given time point and not across time. For instance, patients may progress from any OS level directly to OS 11 (death). This feature allows for the complexity of the relationships in the OS to be considered and modeled directly when considering progression over time. Furthermore, the OS scale is based on procedure, medication, and concept codes commonly found in EHR data. New codes may be added when clinically justified and as new treatments become available. This feature makes the proposed OS scalable to EHR data studies that use the OMOP CDM beyond N3C.

Limitations of this study include the fact that because N3C data are de-identified, chart reviews for patients in the enclave are not possible. Further, since data were entered for patient care rather than research purposes at multiple institutions using different policies and practices, the data dictionary is somewhat limited. For example, it was not possible to verify that a patient received a prescribed drug, such as epinephrine, or to determine exactly why it was prescribed, for example, whether it was given as a vasopressor or local injection. However, this is a limitation inherent in any large multi-center study utilizing de-identified data and should be considered by investigators for all such analyses. Another challenge of utilizing de-identified data is the lack of certainty about the timing of events. For example, many sites associated with N3C report dates that are obfuscated by approximately 7 days. When considering temporal information, such as month or quarter, appropriate care must be taken. To mitigate this impact, we consider the possible date ranges that have been provided to allow researchers and clinicians to understand the variability that comes with timing of events.

Finally, because accurate measurement of the transition between OS level depends on the availability of adequate data, defining longer lengths of time between transitions might increase the likelihood of detecting relevant procedures that define a change in OS level. For instance, our use of a 28-day observation period allows daily visibility into the transitions that occur over a 28-day period. However, if information on a concept was not noted in any of the 28 days, we assumed the patient remained at OS 1 for the entire period.

## CONCLUSION

The presented OS provides a useful framework for the evaluation of COVID-19 patient severity status using multi-site EHR data from across the United States. An OS for scoring the severity of SARS-CoV-2 infection over time using the OMOP/OHDSI environment enables understanding of the clinical courses of large numbers of SARS-CoV-2-infected patients and may potentially support demonstration of therapeutic associations with improved clinical status. The ability to have high fidelity in predicting outcomes among subsets of COVID-19 patients is facilitated by the very large numbers available in EHR datasets such as N3C. Future extension of this work may inform treatment decisions and may enable better prediction of staffing, patient census, and resource allocation.

## FUNDING

Research in this publication was supported by the National Institute of General Medical Sciences of the National Institutes of Health under Award Number 5U54GM104942-05S2 and 5U54GM104942-06S4. The content is solely the responsibility of the authors and does not necessarily represent the official views of the National Institutes of Health. The funders had no role in the study design, data collection and analysis, nor any preparation of any content of the manuscript.

## AUTHOR CONTRIBUTIONS

MK, BSP, and HTB designed and performed the analyses for the study. MK, JH, and AJA provided concept set development. MK and HTB provided code for the ordinal scale development. JZP, SLH, and MTV created the ordinal scale structure and translation from WHO model. SLH, JZP, MTV provided clinical guidance. All authors contributed to write the draft and provided feedbacks. SLH supervised the team.

## ETHICS APPROVAL

National Institute of Health’s National COVID Cohort Collaborative Data Utilization Request Approval committee approved the data utilization request of this project (RP-504BA5). Each author’s home Institutional Review Board approved the study protocol (MK, BSP, WDK, BH, and SLH # 2012192778; HTB # 1700991; AJA and JH # 050-21- EP; HM # 2020V0280; SLS # 1697848-2).

## Supplementary Material

ooac066_Supplementary_Data

## Data Availability

Procedure concepts, raw SQL/R code developed in this study, and summary data are available in Dryad Digital Repository, at https://doi.org/10.5061/dryad.dncjsxm2q and GitHub repository, https://github.com/National-COVID-Cohort-Collaborative/CS-Rural-Health/tree/main/ordinal-scale-EHR. N3C is a public resource maintained by NCATS to support COVID-19 research. Investigators can request access to the Enclave here.
