# Supplementary material for: An ordinal severity scale for COVID-19 retrospective studies using Electronic Health Record data
Source: JAMIA Open. 2022 Jul 9;5(3):ooac066. doi: 10.1093/jamiaopen/ooac066 (PMC9278199; doi:10.1093/jamiaopen/ooac066)

Supplementary Material

Data available from the Dryad Digital Repository: https://doi.org/10.5061/dryad.dncjsxm2q.

Table of content

Table A1: Summary of Oxygen (OS=5) EHR **records** by concept name ……………..……………………………………………….2
Table A2: Summary of ventilation (OS =7) EHR **records** by concept name ……….…………………...……………………………3
Table A3: Summary of ECMO or vasopressor use (OS =9) EHR **records** by concept name ………………………..………………4

Figure 4- Ribbon chart for hospitalized patients over 28-day ………………………………….……………………………………..5

Table A1: Summary of Oxygen (OS=5) EHR records by concept name

	CC1, N(%)	CC2, N(%)	CC3, N(%)	CC4, N(%)	CC5, N(%)	CC6, N(%)	CC7, N(%)	CC8, N(%)	Other, N(%)
**Week**									
Week 1	453,694 (55%)	95,030 (59%)	41,931 (39%)	26,954 (50%)	13,928 (31%)	24,225 (71%)	9,226 (75%)	5,444 (78%)	13,204 (75%)
Week 2	221,973 (27%)	39,348 (24%)	31,439 (29%)	16,244 (30%)	13,974 (31%)	6,058 (18%)	1,977 (16%)	979 (14%)	2,744 (16%)
Week 3	96,344 (12%)	16,964 (11%)	20,704 (19%)	7,051 (13%)	10,326 (23%)	2,509 (7%)	716 (6%)	361 (5%)	1,059 (6%)
Week 4	53,287 (6%)	9,518 (6%)	12,947 (12%)	3,545 (7%)	6,812 (15%)	1,104 (3%)	416 (3%)	195 (3%)	605 (3%)
**Min quarter of dx**									
2020-Q2	86,697 (11%)	27,569 (17%)	27,805 (26%)	4,899 (9%)	9,402 (21%)	3,991 (12%)	0 (0%)	774 (11%)	874 (5%)
2020-Q3	116,955 (14%)	15,523 (10%)	8,683 (8%)	5,039 (9.4%)	3,732 (8%)	3,813 (11%)	77 (0.6%)	710 (10%)	619 (3.5%)
2020-Q4	273,512 (33%)	55,110 (34%)	23,197 (22%)	19,768 (37%)	13,721 (30%)	10,334 (30%)	2,902 (24%)	2,062 (30%)	4,419 (25%)
2021-Q1	177,279 (21%)	28,514 (18%)	13,182 (12%)	9,659 (18%)	6,273 (14%)	7,191 (21%)	2,481 (20%)	1,833 (26%)	3,541 (20%)
2021-Q2	47,724 (5.8%)	11,390 (7.1%)	8,549 (8.0%)	5,625 (10%)	6,081 (14%)	3,848 (11%)	2,569 (21%)	711 (10%)	3,285 (19%)
2021-Q3	109,332 (13%)	17,524 (11%)	14,400 (13%)	8,306 (15%)	1,330 (3%)	4,405 (13%)	4,306 (35%)	853 (12%)	4,751 (27%)
**Max quarter of dx**									
2020-Q2	86,697 (11%)	27,569 (17%)	27,806 (26%)	4,767 (9%)	9,402 (21%)	3,983 (12%)	0 (0%)	769 (11%)	850 (5%)
2020-Q3	116,955 (14%)	15,523 (10%)	8,683 (8%)	4,941 (9%)	3,732 (8%)	3,817 (11%)	76 (0.6%)	708 (10%)	636 (4%)
2020-Q4	273,512 (33%)	55,110 (34%)	23,197 (22%)	19,343 (36%)	13,721 (30%)	10,271 (30%)	2,893 (23%)	2,048 (29%)	4,396 (25%)
2021-Q1	177,279 (21%)	28,514 (18%)	13,182 (12%)	10,084 (19%)	6,273 (14%)	7,233 (21%)	2,491 (20%)	1,833 (26%)	3,565 (20%)
2021-Q2	47,724 (5.8%)	11,390 (7%)	8,549 (8%)	5,684 (11%)	6,081 (14%)	3,862 (11%)	2,567 (21%)	721 (10%)	3,284 (19%)
2021-Q3	109,332 (13%)	17,524 (11%)	14,400 (13%)	8,459 (16%)	1,330 (3%)	4,421 (13%)	4,308 (35%)	863 (12%)	4,761 (27%)

CC1: Delivered oxygen flow rate
CC2: Inhaled oxygen flow rate
CC3: Inhaled oxygen concentration
CC4: Continuous positive airway pressure ventilation (CPAP), initiation and management
CC5: Oxygen gas flow Oxygen delivery system
CC6: Assistance with Respiratory Ventilation, Less than 24 Consecutive Hours, Continuous Positive Airway Pressure
CC7: Assistance with Respiratory Ventilation, Less than 24 Consecutive Hours, High Nasal Flow/Velocity
CC8: Assistance with Respiratory Ventilation, 24-96 Consecutive Hours, Continuous Positive Airway Pressure
Others: All other procedures/concept names with less than 5000 events

All numbers represent counts of events not patients.

Table A2: Summary of ventilation (OS =7) EHR records by concept name

	CC1, N(%)	CC2, N(%)	CC3, N(%)	CC4, N(%)	CC5, N(%)	CC6, N(%)	CC7, N(%)	CC8, N(%)	CC9, N(%)	CC10, N(%)	CC11, N(%)	CC12, N(%)	Other, N(%)
**Week**													
Week 1	85,631 (40%)	32,380 (27%)	36,343 (31%)	29,940 (67%)	22,140 (62%)	13,052 (60%)	8,209 (58%)	9,779 (67%)	7,012 (62%)	3,548 (36%)	4,208 (83%)	8,093 (34%)	85,631 (40%)
Week 2	55,261 (26%)	30,570 (25%)	36,176 (31%)	9,256 (21%)	8,363 (23%)	3,765 (17%)	3,377 (24%)	2,751 (19%)	2,368 (21%)	2,844 (29%)	470 (9%)	4,778 (20%)	55,261 (26%)
Week 3	41,368 (19%)	29,703 (25%)	27,311 (23%)	4,038 (9.0%)	3,801 (11%)	2,900 (13%)	1,653 (12%)	1,341 (9%)	1,226 (11%)	2,022 (21%)	259 (5.1%)	5,551 (24%)	41,368 (19%)
Week 4	30,992 (15%)	28,494 (24%)	18,581 (16%)	1,621 (4%)	1,472 (4%)	1,891 (9%)	1,036 (7%)	685 (5%)	704 (6%)	1,419 (14%)	142 (3%)	5,176 (22%)	30,992 (15%)
**Min quarter of dx**													
2020-Q2	55,000 (26%)	37,207 (31%)	26,012 (22%)	8,941 (20%)	7,034 (20%)	4,657 (22%)	3,476 (24%)	3,135 (22%)	2,092 (18%)	3,932 (40%)	785 (15%)	5,715 (24%)	55,000 (26%)
2020-Q3	10,028 (5%)	5,300 (4%)	12,207 (10%)	5,075 (11%)	3,847 (11%)	1,942 (9.0%)	1,543 (11%)	1,361 (9%)	1,318 (12%)	394 (4%)	546 (11%)	2,596 (11%)	10,028 (5%)
2020-Q4	63,383 (30%)	25,759 (21%)	28,942 (24%)	10,623 (24%)	8,543 (24%)	5,057 (23%)	3,403 (24%)	3,696 (25%)	2,725 (24%)	1,644 (17%)	1,382 (27%)	5,023 (21%)	63,383 (30%)
2021-Q1	40,448 (19%)	27,552 (23%)	18,844 (16%)	8,211 (18%)	7,230 (20%)	3,723 (17%)	1,993 (14%)	2,524 (17%)	2,275 (20%)	2,041 (21%)	965 (19%)	4,401 (19%)	40,448 (19%)
2021-Q2	16,449 (8%)	14,905 (12%)	11,371 (9.6%)	4,027 (9.0%)	2,432 (6.8%)	1,674 (7.7%)	1,269 (9%)	1,248 (9%)	860 (8%)	972 (10%)	468 (9%)	2,219 (9%)	16,449 (8%)
2021-Q3	9,257 (4%)	1,946 (2%)	15,388 (13%)	4,882 (11%)	3,980 (11%)	2,731 (13%)	1,512 (11%)	1,935 (13%)	1,386 (12%)	569 (6%)	839 (17%)	2,029 (9%)	9,257 (4.3%)
**Max quarter of dx**													
2020-Q2	4,647 (22%)	3,515 (25%)	2,812 (19%)	2,107 (19%)	3,932 (40%)	785 (15%)	5,639 (24%)	4,647 (22%)	3,515 (25%)	2,812 (19%)	2,107 (19%)	3,932 (40%)	785 (15%)
2020-Q3	1,920 (9%)	1,554 (11%)	1,660 (11%)	1,316 (12%)	394 (4.0%)	546 (11%)	2,767 (12%)	1,920 (9%)	1,554 (11%)	1,660 (11%)	1,316 (12%)	394 (4%)	546 (11%)
2020-Q4	5,001 (23%)	3,387 (24%)	3,670 (25%)	2,715 (24%)	1,644 (17%)	1,382 (27%)	4,974 (21%)	5,001 (23%)	3,387 (24%)	3,670 (25%)	2,715 (24%)	1,644 (17%)	1,382 (27%)
2021-Q1	3,737 (17%)	2,011 (14%)	2,539 (17%)	2,286 (20%)	2,041 (21%)	965 (19%)	4,442 (19%)	3,737 (17%)	2,011 (14%)	2,539 (17%)	2,286 (20%)	2,041 (21%)	965 (19%)
2021-Q2	1,671 (8%)	1,269 (9%)	1,270 (9%)	863 (8%)	972 (10%)	468 (9%)	2,240 (9%)	1,671 (8%)	1,269 (8.9%)	1,270 (9%)	863 (8%)	972 (10%)	468 (9%)
2021-Q3	2,836 (13%)	1,516 (11%)	1,963 (13%)	1,392 (12%)	569 (5.8%)	839 (17%)	2,038 (9%)	2,836 (13%)	1,516 (11%)	1,963 (13%)	1,392 (12%)	569 (6%)	839 (17%)

CC1: Intubation, endotracheal, emergency procedure
CC2: Tracheostomy, planned (separate procedure)
CC3: Ventilation assist and management, initiation of pressure or volume preset ventilators for assisted or controlled breathing; hospital inpatient/observation, each subsequent day
CC4: Respiratory Ventilation, Greater than 96 Consecutive Hours
CC5: Insertion of Endotracheal Airway into Trachea, Via Natural or Artificial Opening
CC6: Dependence on respirator
CC7: Insertion of Endotracheal Airway into Trachea, Via Natural or Artificial Opening Endoscopic
CC8: Ventilation assist and management, initiation of pressure or volume preset ventilators for assisted or controlled breathing; hospital inpatient/observation, initial day
CC9: Respiratory Ventilation, 24-96 Consecutive Hours
CC10: Ventilator finding
CC11: Artificial respiration
Others: All other procedures/concept names with less than 5000 events

All numbers represent counts of events not patients.

Table A3: Summary of ECMO (OS =9) EHR records by concept name

	CC1, N(%)	CC2, N(%)	CC3, N(%)	CC4, N(%)	CC5, N(%)	CC6, N(%)	Other, N(%)
**Week**							
Week 1	14,131 (23%)	8,563 (23%)	8,300 (23%)	2,411 (19%)	2,543 (19%)	1,818 (23%)	4,897 (34%)
Week 2	17,082 (27%)	10,309 (28%)	9,702 (27%)	3,608 (28%)	4,048 (30%)	2,428 (31%)	4,252 (30%)
Week 3	16,519 (27%)	9,600 (26%)	9,695 (27%)	3,801 (30%)	3,780 (28%)	1,817 (23%)	2,988 (21%)
Week 4	14,316 (23%)	8,314 (23%)	8,653 (24%)	3,164 (24%)	3,114 (23%)	1,722 (22%)	2,252 (16%)
**Min quarter of dx**							
2020-Q2	15,771 (27%)	9,471 (27%)	10,473 (30%)	2,156 (19%)	5,543 (44%)	238 (3%)	16,883 (50%)
2020-Q3	2,584 (4%)	158 (0.5%)	185 (0.5%)	16 (0.1%)	70 (0.6%)	99 (1%)	4,092 (12%)
2020-Q4	7,934 (13%)	4,033 (12%)	4,056 (11%)	3,635 (33%)	787 (6%)	353 (5%)	1,527 (5%)
2021-Q1	13,822 (23%)	9,755 (28%)	9,757 (27%)	1,825 (16%)	2,468 (19%)	299 (4%)	1,081 (3%)
2021-Q2	11,572 (19%)	7,596 (22%)	6,194 (17%)	2,377 (21%)	1,402 (11%)	3,860 (57%)	3,029 (9%)
2021-Q3	6,611 (11%)	1,946 (6%)	3,314 (10%)	26 (0.2%)	901 (7%)	1,455 (21%)	6,418 (19%)
**Max quarter of dx**							
2020-Q2	16,745 (27%)	11,754 (32%)	11,298 (31%)	3,625 (28%)	7,838 (58%)	1,677 (22%)	7,710 (32%)
2020-Q3	2,462 (4%)	162 (0.4%)	185 (0.5%)	15 (0.1%)	70 (0.5%)	128 (2%)	4,092 (17%)
2020-Q4	8,073 (13%)	4,032 (11%)	4,054 (11%)	3,637 (28%)	787 (6%)	353 (5%)	1,526 (6%)
2021-Q1	13,722 (22%)	9,747 (26%)	9,753 (27%)	1,823 (14%)	2,463 (18%)	229 (3%)	1,019 (4%)
2021-Q2	11,615 (19%)	7,600 (21%)	6,197 (17%)	2,377 (18%)	1,403 (10%)	3,917 (50%)	3,085 (13%)
2021-Q3	6,702 (11%)	1,957 (5%)	3,319 (9%)	28 (0.2%)	905 (7%)	1,468 (19%)	6,446 (27%)

CC1: Extracorporeal membrane oxygenation (ECMO)/extracorporeal life support (ECLS) provided by physician; daily management, each day, veno-venous
CC2: Extracorporeal membrane oxygenation (ECMO)/extracorporeal life support (ECLS) provided by physician; insertion of peripheral (arterial and/or venous) cannula(e), percutaneous, 6 years and older (includes fluoroscopic guidance, when performed)
CC3: Extracorporeal membrane oxygenation (ECMO)/extracorporeal life support (ECLS) provided by physician; initiation, veno-venous
CC4: Extracorporeal membrane oxygenation (ECMO)/extracorporeal life support (ECLS) provided by physician; reposition peripheral (arterial and/or venous) cannula(e), percutaneous, 6 years and older (includes fluoroscopic guidance, when performed)
CC5: Extracorporeal membrane oxygenation (ECMO)/extracorporeal life support (ECLS) provided by physician; removal of peripheral (arterial and/or venous) cannula(e), percutaneous, 6 years and older
CC6: Extracorporeal membrane oxygenation (ECMO)/extracorporeal life support (ECLS) provided by physician; daily management, each day, veno-arterial
Others: All other procedures/concept names with less than 5000 events

All numbers represent counts of events not patients.

Figure A1- Ribbon chart for hospitalized patients over 28-day

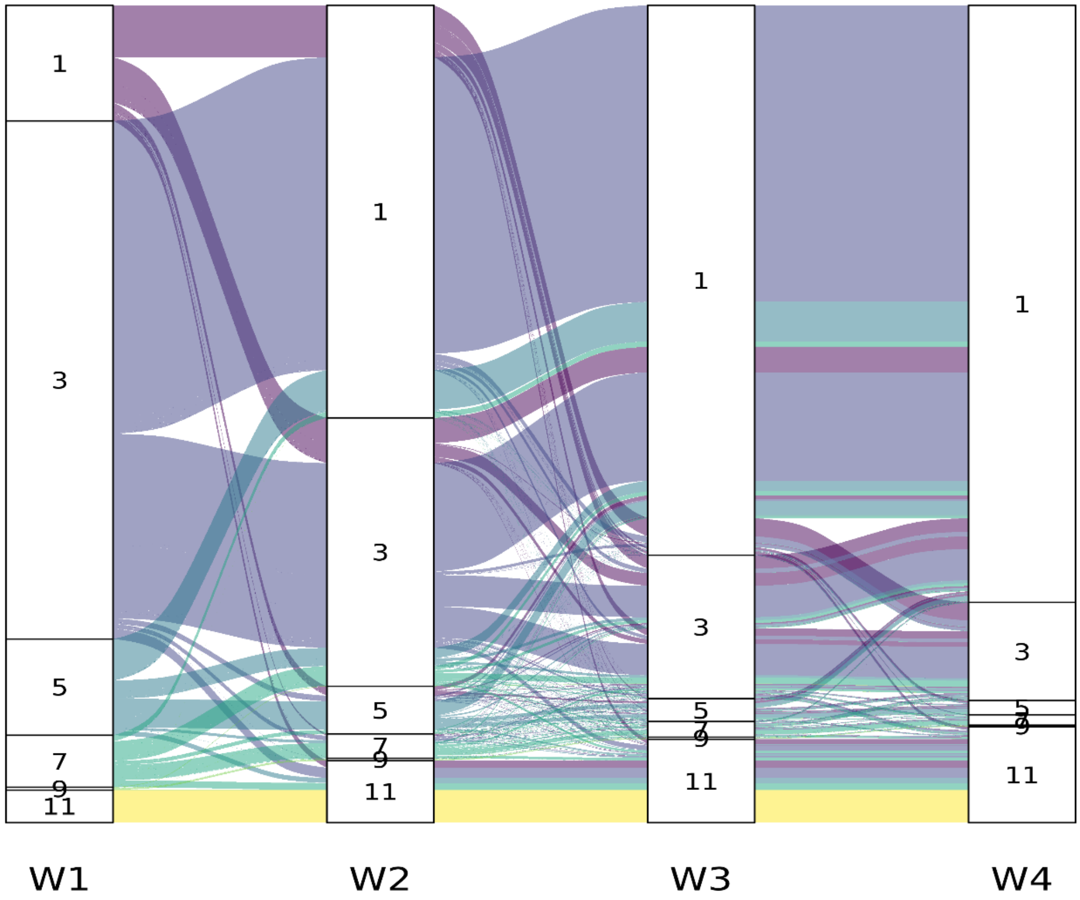

Supplement: ooac066_Supplementary_Data [file ooac066_supplementary_data.pdf]
